# Genetic engineering and genome editing technologies as catalyst for Africa’s food security: the case of plant biotechnology in Nigeria

**DOI:** 10.3389/fgeed.2024.1398813

**Published:** 2024-07-09

**Authors:** Muyiwa Seyi Adegbaju, Titilayo Ajose, Ifeoluwa Elizabeth Adegbaju, Temitayo Omosebi, Shakirat Oloruntoyin Ajenifujah-Solebo, Olaitan Yetunde Falana, Olufunke Bolatito Shittu, Charles Oluwaseun Adetunji, Olalekan Akinbo

**Affiliations:** ^1^ Department of Crop, Soil and Pest Management, Federal University of Technology Akure, Akure, Ondo, Nigeria; ^2^ Fruits and Spices Department, National Horticultural Institute, Ibadan, Oyo, Nigeria; ^3^ Department of Agricultural Technology, Federal College of Forestry, Jos, Nigeria; ^4^ Department of Genetics, Genomic and Bioinformatics, National Biotechnology Research and Development Agency, Abuja, Nigeria; ^5^ Department of Microbiology, College of Biosciences, Federal University of Agriculture, Abeokuta, Nigeria; ^6^ Department of Microbiology, Edo State University, Uzairue, Edo, Nigeria; ^7^ African Union Development Agency-NEPAD, Office of Science, Technology and Innovation, Midrand, South Africa

**Keywords:** biotechnology policy, crop improvement, molecular farming, transgenic crops, biotechnology adoption

## Abstract

Many African countries are unable to meet the food demands of their growing population and the situation is worsened by climate change and disease outbreaks. This issue of food insecurity may lead to a crisis of epic proportion if effective measures are not in place to make more food available. Thus, deploying biotechnology towards the improvement of existing crop varieties for tolerance or resistance to both biotic and abiotic stresses is crucial to increasing crop production. In order to optimize crop production, several African countries have implemented strategies to make the most of this innovative technology. For example, Nigerian government has implemented the National Biotechnology Policy to facilitate capacity building, research, bioresource development and commercialization of biotechnology products for over two decades. Several government ministries, research centers, universities, and agencies have worked together to implement the policy, resulting in the release of some genetically modified crops to farmers for cultivation and Commercialization, which is a significant accomplishment. However, the transgenic crops were only brought to Nigeria for confined field trials; the manufacturing of the transgenic crops took place outside the country. This may have contributed to the suspicion of pressure groups and embolden proponents of biotechnology as an alien technology. Likewise, this may also be the underlying issue preventing the adoption of biotechnology products in other African countries. It is therefore necessary that African universities develop capacity in various aspects of biotechnology, to continuously train indigenous scientists who can generate innovative ideas tailored towards solving problems that are peculiar to respective country. Therefore, this study intends to establish the role of genetic engineering and genome editing towards the achievement of food security in Africa while using Nigeria as a case study. In our opinion, biotechnology approaches will not only complement conventional breeding methods in the pursuit of crop improvements, but it remains a viable and sustainable means of tackling specific issues hindering optimal crop production. Furthermore, we suggest that financial institutions should offer low-interest loans to new businesses. In order to promote the growth of biotechnology products, especially through the creation of jobs and revenues through molecular farming.

## Introduction

The human population, which is presently about 8 billion, has been projected to rise drastically to 10.4 billion by the end of 21st century (www.ourworldindata.org). Although this is a significant milestone for the planet Earth, there is still much work to be done to balance the exponential growth in population and human requirements for clothing, food, water, and safety. In the last fifty years, significant progress has been made in science and technology. Emergence of new field of research in molecular biology and molecular genetics collectively referred to as “Omics” has created endless possibilities for biotechnological applications in various aspects of human life. The impact of biotechnology is already being felt, particularly in the development and production of effective vaccines against communicable and non-communicable diseases. Also, through innovative ideas, coupled with advancements in modern biotechnology techniques, some major food crops have been made healthier by the alteration of the quality or content of their main nutrients (Zhao et al., 2021). In the coming years, the impact of climate change in agriculture will become more aggravated, especially in Africa, where the yield per unit area of crops grown is already the lowest globally (Kyetere et al., 2019). Thus, application of plant biotechnology techniques has been recommended for improved food productivity, through the acceleration of the development of new crop varieties with better capacity for high yield (Lloyd et al., 2023).

This paper discusses the economic impact of biotechnology sector in developed and developing countries around the world, explaining the strategies being used in places where it is being fully explored. Although biotechnology is still perceived by many as an emerging technology that poses a risk to human health and environment, progress made so far in adopting its tools as catalyst for food security in Africa were examined as well as policies put in place by many countries in the world, especially in Africa to regulate crops developed through its application (AUDA-NEPAD APET Genome Editing Policy Framework, AAGEPF, 2022). It is our opinion that certain factors, limiting Africa from reaching its full potential in agricultural production can be addressed by smart combination of biotechnological and conventional breeding approaches. To attain food sufficiency in Nigeria, we highlight key areas where biotechnological techniques can be deployed to increase production of important crops in the country. An instance of a campaign against the development of genetically modified crops in Nigeria and possible solutions to prevent the re-occurrence of such in the future was presented and discussed. In light of the aforementioned, this review uses Nigeria and the possibility of improving its common crops using this technology as a case study to demonstrate how cutting-edge biotechnology, such as genetic engineering and genome editing, can be used to expedite the process of ensuring food security in the continent of Africa.

## Biotechnology and food security in Africa

The global market value of biotechnology stands at 295 billion USD. In 2019, the industry successfully employed approximately 900,000 people (Martin et al., 2021). The area has given rise to four well-established technological paths: industrial, medicinal, agricultural, and environmental biotechnology. These sectors are mainly concentrated in developed countries, with USA leading in terms of investment. Since the year 2005, there has been a constant global increase in agricultural biotechnology. Africa, through the African Union (AU), has noticed rapid advancement in biotechnology and has made efforts to secure access to emerging technology in the field. This was demonstrated when the organisation constituted the African Panel on Emerging Technologies (APET) in 2016. Some of the roles of the high level-APET is to provide advice on most the rational approach, strategy and policy regulations for emerging biotechnological techniques in Africa (AAGEPF, 2022) and to advise the union and member states on how to harness emerging biological technology towards agricultural productivity and economic development of the continent.

Africa is considered the epicentre of anaemia and micronutrient-deficient people in the world, with children under the age of five years, adolescent girls, and women being mostly affected. Many people on the continent have become food insecure because of conflicts, cultural discrimination, extreme weather events, as well as poverty and economic shocks (Stevens et al., 2022). This situation has highlighted the failure of current economic models at address the development challenges that the developing world faces. Concomitantly, some other challenges, associated with natural resource constraints, such as insufficiency of water and arable land, which in turn has resulted in an increased rate of unemployment, poverty, and inequality are also being experienced in this part of the world (Oxfam, 2019; Harris-Fry et al., 2020; UNICEF, 2023). Particularly, the poor in rural areas are the most vulnerable and affected, with approximately 88.4 million Nigerians living in extreme poverty and under-development (Statista, 2024).

In 2019, Africa spent 43 billion USD on food importation, which is forecast to hit 90 billion USD by 2030. Decline in per capita food production on the continent is partly due to population explosion which is not matched up with adequate food production. This has resulted in widening the gap between food production and the associated consumption, according to Africa Common Position on Food Systems Food Security (ACPOFS, 2021). Currently, Nigeria is a food deficit nation, spending 10 billion USD annually on food importation to feed its ever-growing population. Although the country leads globally in the production of crops like cassava, yam, and taro, this is mostly due to the annual increase in land area under cultivation but not as a result of improved productivity, in terms of yield/ha (Ikuemonisan et al., 2020; Fufa et al., 2023; Kalu et al., 2023). Even so, post-harvest yield loss (up to 60% in some crops) occurs at various stages of food system, thereby making food unaffordable and unavailable to many (Morris et al., 2019; Ewa, 2021; Businessday NG, 2023). To meet the food needs of its people and ensure food security, Nigeria must embrace novel technology in agriculture and overhaul the food system completely.

## Important techniques of biotechnology for food security

**FIGURE 1:**
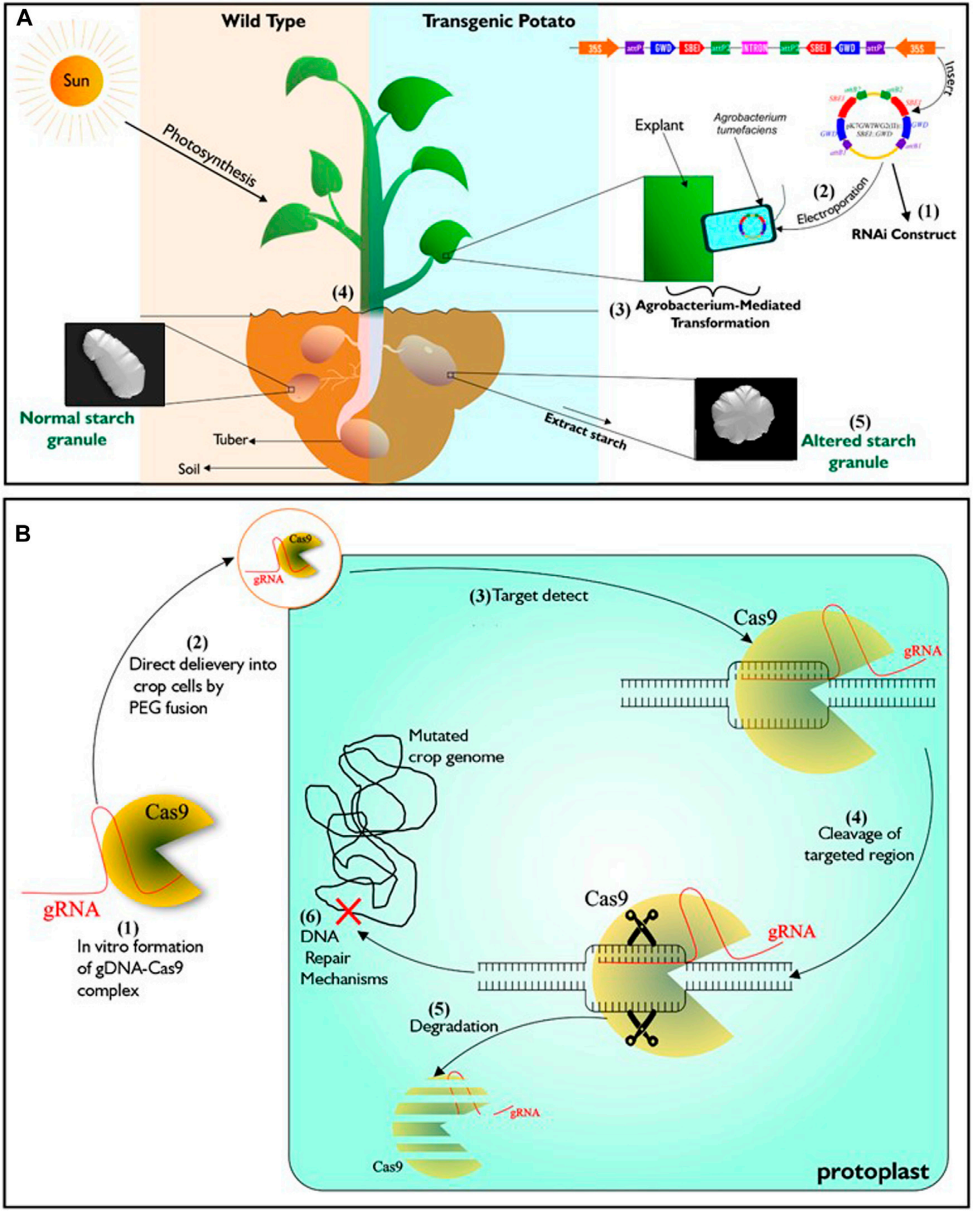
Illustration of genetic engineering (RNAi) and DNA-free CRISPR/Cas9. **(A)** Described RNAi gene silencing technology. It starts with the construction of T-Plasmid which contains the coding sequence of the targeted gene/s and antibiotics resistance gene. *Agrobacterium tumefaciens* is then transformed with the RNAi construct which is then used in transforming young plant tissues such as leaves or internode from tissue cultured wild type. To confirm successful transformation events, putative transgenic lines that are resistant to the incorporated antibiotics resistant genes are selected. Transgenic lines will exhibit phenotypes that is different from wild type depending on the function of the silenced genes. **(B)** Described DNA-free CRISPR/Cas9 technology. It starts with *in vitro* complex formation between recombinant Cas9 and gRNA. Then the CRISPR/Cas ribonucleoprotein (RNP) complex is directly delivered to protoplasts by PEG fusion. After delivery into the protoplast, The RNP complex is active and enabled to precisely locate the target genomic region/s to induce double strand breaks. Mutation in the genome occurs after inherent cell repairs at the targeted genomic regions without the addition of foreign DNA. Subsequently, complete degradation of RNP complex occurs within the cell.

Genetic engineering is one of such advanced technologies that is being utilized to attain food sufficiency in developed countries of the world. It involves exploring knowledge of the functional genomics of species and organisms, by incorporating specific DNA sequences coding for desirable traits into crops of interest (Chen et al., 2017; Narayanan et al., 2019), using tools of gene transfer such as *Agrobacterium*-mediated transformation, protoplast transformation, electroporation, particle bombardment and calcium-phosphate-mediated gene transformation. This technology can, in addition, bring about silencing (Figure 1A), over-expression or complete loss of function of a specific gene within plants (Brummell et al., 2015; Ferreira et al., 2017; Wang et al., 2018; Zhong et al., 2019). Crops developed through this approach are referred to as transgenic or genetically modified organism (GMO). A meta-analysis of data from the maize field by Pellegrino et al. (2018) over a period of 21 years indicated that genetically engineered maize performed better in grain yield than those from near-isogenic lines.

Genome editing (GEd) technology is another aspect of biotechnology currently experiencing growth. It enables scientists to manipulate the genome (the entire genetic information found in a cell) of various living organisms. It is based on the use of engineered sequence specific nucleases (SSNs) to induce DNA double-stranded breaks at precise locus/loci in the genome, followed by repair through either of the two cellular DNA repair pathways, including error-prone non-homologous end joining (NHEJ) or homology directed repair (HDR) (Wyman and Kanaar, 2006; Malzahn et al., 2017). Zinc finger nucleases (ZFNs) (Kim et al., 1996), homing endonucleases (HE) or Meganucleases (MN) (Chevalier and Stoddard, 2001) and transcription activator-like effector nucleases (TALEN) (Boch et al., 2009) are the three distinct SSNs, engineered prior to the emergence of CRISPR-CaS system, the most recent technique and by far the most popular tool for genome editing (Wiedenheft et al., 2012). Unlike transgenic plants, a major feature of the genome editing is the ability to alter a plant’s genome without incorporating foreign DNA (Figure 1B; Malzahn et al., 2017). Application of genome editing in crop improvement is favoured for its cost-effectiveness, ease of use and possibility of speeding up the development of elite varieites.

## Regulation of GMO and GEd crops and products

There are still unanswered questions about the safety of genetically modified crops for humans and the environment, despite efforts to maximize the benefits of genetic engineering for food security. Because of this, most nations have implemented or are implementing regulatory frameworks that cover the creation, processing, and use of genetically modified organisms (GMOs), though the specifics of these frameworks vary from one place to another. This is because regulators in diverse countries either take a product-based approach or process-based approach for safety regulation of GM crops. A product-base approach assesses risks and gains of GM crop on a case and case basis, a process-based approach the assesses method used in producing GM crops. For example, regulators of GMO in the EU, Australia and New Zealand are concerned about uncertainties in the technology and have refused to approve most GMO products. These items are labelled on the shelf even after they are approved, giving customers flexibility in their selection. This is in sharp contrast to GMO regulation in countries like the United States, Japan, Mexico, and Canada, where regulators ruled that the technology is safe, hence, the approval of products obtained there from for production and consumption. Labelling of GM products in these countries is only done when the nutritional and compositional content is altered or possesses new allergens (Buchholz and Collins, 2010).

The debate over crop enhancement through the use of genome editing (GEd) technology is growing in popularity, as is the use of its products. The public perception did not favour the utilization of GMOs and its regulations are very strict in many countries. Most countries employ science-based risk analysis in regulating such products. The rational approach proposed by scientists for regulating GEd crops is one which designates as GMO, any plant with foreign DNA inserted into its genome whereas any, with no insertion of such is regulated in the same manner as variety developed through conventional breeding methods (Lema, 2019). For example, the approach for regulating GEd crops by biosafety regulators in countries like Australia, Argentina, Brazil, Chile, Canada, United States and Japan, is the same as those that apply to conventional varieties, if foreign DNA or genes are not integrated into the genome (Lema, 2019; Hundleby and Harwood, 2022). Regulations, adopted by Australia for GEd plant is similar to that in Argentina. However, in New Zealand, despite the ruling on environmental protection authority that GEd plants lacking foreign DNA should not be regulated as GMOs in 2014; this stance was later revoked by High Court and currently, GEd crops are categorised as GMOs (Fritsche et al., 2018).

In Africa, Nigeria was the first country to make the move to amend its biosafety legislation to include regulation of genome-edited products (AAGEPF, 2022; AUDA-NEPAD APET Genome Editing Policy Framework, 2022**)**. Thereafter, the guidelines for regulating GEd products were clearly stated and have been adopted since 2020. Other African nations, including Burkina Faso, Ethiopia, Ghana, Kenya, and Malawi, followed suit and enacted national rules for genetically engineered products. The named countries, however exempted regulation of genome-edited crops with no foreign DNA from their biosafety laws. The move is adjudged positive as it will facilitate increase in agricultural productivity of these countries. South Africa still classifies all GEd plants as GM crops, in contrast to other African nations like eSwatini, Senegal, Mozambique, Namibia, Rwanda, Togo, Zambia, Zimbabwe and others who have expressed interest in creating laws for genome-edited products. However, experts opine that such approach contradict the principle of science-based risk analysis and the decision is being appealed (The conversation, 2022).

## Involvement of governments in plant biotechnology

The United States of America has continuously led the world in biotechnology research and development as well as the commercialization of its goods for more than 20 years. The United States government implemented regulations in the early years of biotechnology to encourage university-based biotechnology research and development. In 1986, US government enacted the Federal Technology Transfer Act, to foster transfer of publicly developed techniques in biotechnology to private-enterprise (Mugabe et al., 2002). The law made room for joint research between federal laboratories, universities, and private-enterprises and at the inception of innovation, the private partner acquires the patent rights while the participating university and/or government innovators share royalties from licensed innovation. As far back as 1987, the US government spent 2.7 billion USD as funding for biotechnology research and later increased to three billion USD annually by mid 1990s (Avramovic, 1996).

Early investment in R&D and good policy may have encouraged private-sector participation in development and commercialization of biotechnology products and may also have been responsible for huge revenue now generated from their sales. Presently, the US generates a revenue of 33 billion USD from its 318 biotechnology companies (Martin et al., 2021). Because the industry is extremely technical, US government is still committed to funding R&D and training in biotechnology. However, the burden of financing scientific research in the field seems to have shifted to the private-enterprises, as 70% of the funding now comes from that sector. This trend has also been reported in other countries like France and Japan (Martin et al., 2021), an indication that biotechnology sector is highly profitable.

**FIGURE 2:**
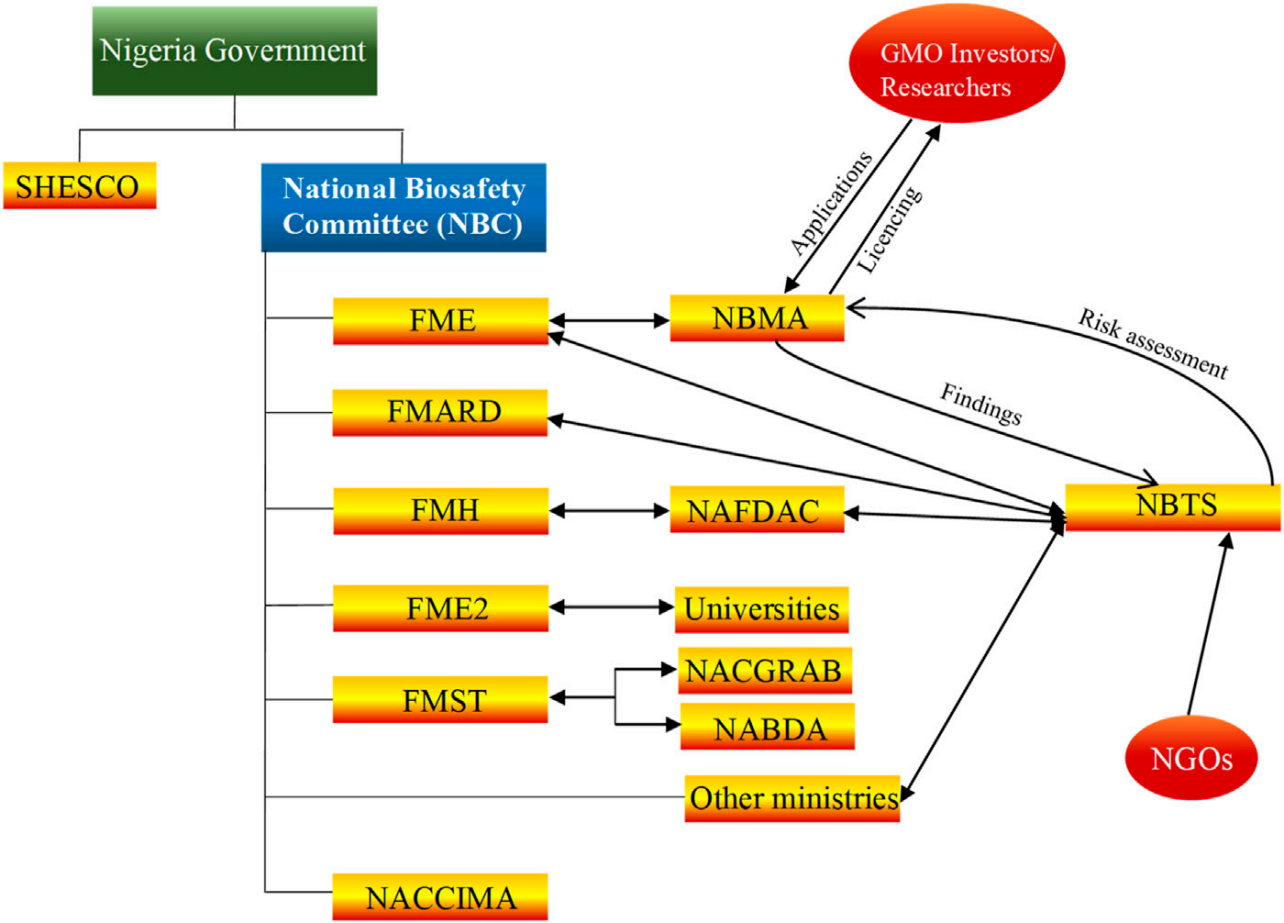
An overview of the biotechnology strategy in Nigeria. Many government agencies and ministries are involved in setting up, implementation of biotechnology policy and regulation of biotech products in Nigeria. Those that are most relevant in the implantation and regulation of biotechnology are National Biotechnology Development Agency (NABDA), National Agency for Food and Drug Administration and Control (NAFDAC), National Biosafety Management Agency (NBMA), Federal Ministries, Nigerian Association of Chambers of Commerce, Industry, Mines, and Agriculture (NACCIMA) and The National Centre for Genetic Resources and Biotechnology (NACGRAB), Sheda Science and Technology Complex (SHESTCO). Research institutes and Universities are involved in human and capacity building, the national Vaccine governing committee consisting of research institutions and federal ministry of health are involved in vaccine research, development, and production in Nigeria.

Various developmental strategies were deployed by other countries towards advancement of biotechnology. For example, Brazil gained international prominence in biotechnology after the successful sequencing of the genome of *Xylella fastidiosa*, a pathogen that causes losses of ∼100 million USD in citrus industry. In 1980, a small team of scientists in Cuba produced alpha-interferon within 42 days. This was a major achievement at the time, which instigated Cuban government in 1986 to fund establishment of centre for Genetic Engineering and Biotechnology. Host of other centers which specialized in biomass conversion, animal production and tropical medicine were also established. As at 2015, biotechnology products, mostly pharmaceuticals, ranked second in the list of most important export commodities in (cuba-solidarity.org.uk 2015; León-de la O et al., 2018). South Korea is another country that has become a biotechnology giant. In 1993, their evolution began, when their government developed a national biotechnology plan, to be executed in three phases, with a total investment of 15 billion USD to be provided by both public and private sectors. Their target was to achieve five percent global market share for novel biotechnology products by 2007. In 2001, the Nigeria Government developed the National Policy for Biotechnology which entails biotechnology knowledge acquisition and commercialization, research and development, capacity building, bioresources development, collaboration in bioresources and biotechnology development. This led to the establishment of National Biotechnology Research and Development Agency (NABDA, 2001) in the same year, which is saddled with the responsibility of implementing the national policy on biotechnology. At the time, strategies for implementing the policy included the identification of Sheda Science and Technology Complex SHESTCO at Federal Capital Territory. In addition, premier universities in the six geo-political zones of the country, namely, University of Ibadan in the southwest; University of Nigeria, Nsukka in the southeast; University of Port-Harcourt in the south-south; Ahmadu Bello University in the northwest; University of Maiduguri in the northeast and the University of Jos, in the northcentral were brought on board to provide necessary facility to support research in biotechnology and genetic engineering. Furthermore, a committee, comprising of representative from Ministries of science and technology, agricultural and rural development, environment health, education, etc., directors of research institutes, universities, manufacturer association of Nigeria and National association of Chambers of Commerce Industry, Mine and Agriculture (NACCIMA), was set up to provide technical expertise needed for biotechnology policy implementation in the country. Currently, the strategy for biotechnology integration in Nigeria has not changed much as indicated in (Figure 2). National Biotechnology Policy was targeted towards accelerated technological growth and increasing self-reliance by strengthening capacity of home-based researchers to copy and adapt techniques in biotechnology for national development. Also, it is expected to serve as government blueprint to effectively address its concerns such as food security among others.

Since its founding, NABRDA has coordinated biotechnology-related activities, leading to commercialized products and promoted agricultural biotechnology across the country; However, in 2015, Nigeria passed a biosafety bill, which gave the National Biosafety Management Agency (NBMA) control over biotechnology activities in the country. NBMA had the responsibility of ensuring safe application, handling of genetically modified organisms and products through licence issuance in Nigeria. In 2015, SHESTCO, in collaboration with WAAPP/PPAAO and ARCN, organized workshop for young and upcoming bio-scientists, drawn from various research institutions in the country, to strengthen biotechnology capacity in the country. Similarly, the biotechnology research and development centre at Ebonyi State University, Abakaliki has commenced summer training on tropical plant biotechnology since 2013. Cross River Government, a state in Nigeria, by way of public private participation in biotechnology promotion, partnered with a biotechnology company to jump start production of feedstock on a large scale for its juice processing plant in Calabar free Trade zone.

## Prospect of using plant biotechnology for improvement of major crops

### Rice

Rice is the single, most significant cereal crop consumed globally, simply because it is a convenience food for the growing world population, with the demand for it expected to increase by 25% by 2030 (Naik et al., 2022; Poutanen et al., 2022). The crop has also been developed into a model monocot system for genetic and functional genomic research (Datta, 2004; Jung et al., 2008). Although, Africa has over 130 M ha of arable land that is suitable for rice production, only approximately 10 M ha of it is currently used for that purpose (IRRI, 2024), which indicates that Africa’s potential in rice production is still largely untapped. For example, Nigeria, currently the largest producer of rice in Africa, has a potential land area of about 4.9 million hectares suitable for rice production, but has limited cropping to about 1.7 million ha, with production being constrained by low input, poor crop management techniques and water scarcity (Abiwon et al., 2016). As a result, of the seven million metric tonnes of the crop consumed in the country in 2022, over 1.5 million metric tonnes were imported to meet the shortfall in rice production (The conversation, 2023).

Two cultivated species of rice, *Oryza sativa* (Asian rice) and *Oryza glaberrima* (African rice) are cultivated in Africa (Van Andel, 2010). Asian rice has been steadily cultivated throughout the world, due to its higher production than African rice, according to productivity assessments. However, because of rich reservoir of genes for resistance to several biotic and abiotic stresses, African rice can thrive better in harsh environment (Wambugu et al., 2013). In certain part of Burkina Faso, Rice farmers prefer cultivation of African rice over Asian rice, due to its resilience against severe environments. Previous efforts to create a rice variety with the high yielding potential of Asian rice and resistance traits of African rice led to the development of New Rice for Africa (NERICA) varieties, which were created by combining traditional breeding techniques with biotechnological approaches. However, African rice is still unrivalled by NERICA in adaptability traits like weed competitiveness (Agnoun et al., 2012). This could be because only around 9% of the *O. glaberrima* genome is present in the genomic content of NERICA variants (Ndjiondjop et al., 2008). Additionally, *O. glaberrima* also possesses valuable genes which can contribute to improvement of nutritional quality of rice. For example, the reports of Gayin et al. (2017) and Wambugu et al. (2019) showed that starch granules, which accumulated in the grain endosperm of African rice, has higher amylose content than its Asian counterpart through structural analysis. This indicates that African rice, with its high amylose content, may be a natural source of resistant starch with potential health benefits, particularly in the treatment of type 2 diabetes, a nutrition-related non-communicable disease that has been increasing in Africa recently (Huang et al., 2018).

Contrary to the view of Sarla and Swamy (2005) that *O. glaberrima* should be used as genetic resource material for improving *O. sativa*, it is opined that its yield can be improved directly, in addition to other valuable agronomical and nutritional traits. Grain shattering, frequently caused by lodging, is the major cause of low yield of *O. glaberrima* (Futakuchi et al., 2008). Evaluation of four *O. sativa* cultivars and 20 accessions of *O. glaberima* revealed that yield reduction of the later was due to grain shattering (Ndjiondjop et al., 2018) and this indicates that improving African rice for resistance against seed shattering will improve harvesting efficiency, thereby making African rice less inferior to *O. sativa*. Recently, a novel gene called *Seed Shattering 11* (*SH11*) in African rice was cloned and characterised (Ning et al., 2023). This gene encodes a MYB transcription factor which inhibits the expression of genes involved in lignin biosynthesis. It was shown that a single nucleotide polymorphism mutation in the coding region of *SH11* increased the binding ability to the GH2 promoter and consequently reduced the lignin content in *O. glaberrima*. However, CRISPR-CAS9 mediated knockout of *SH11* reduced seed shattering significantly in *O. glaberrima* (Ning et al., 2023). This indicates that this gene will be a good target for reducing susceptibility of African rice to grain shattering.

Africa faces a problem with food insecurity that goes beyond a shortage of high-yielding cultivars and high demand. The soils in the majority of the farm land used for crop production have been farmed for a long time, making them naturally less fertile. Thus, one of the main obstacles to rice production in Africa is soil nitrogen deficiency, which forces rice producers to rely largely on inorganic fertilizers. The use of inorganic fertilizer indiscriminately contributes significantly to the acceleration of global warming by releasing nitrous oxide into the atmosphere (AATF, 2024). To curb this, scientists in Africa, led by African Agricultural Technology Foundation, have developed Nitrogen Use Efficiency (NUE12) rice variety under the NEWEST Rice project. NUE12 is a transgenic event which involved insertion of barley’s alanine aminotransferase gene (*HvAlaAT*) into the nuclear genome of NERICA-4. The transgenic variety significantly out-performed Wild type (NERICA 4) in terms of yield at varying levels of Nitrogen application and as such, farmers can opt for lower cost (50%) of N fertilizer while maintaining the yield or the same quantity of nitrogen and increase yield. Three countries, namely; Uganda, Ghana and Nigeria are currently implementing processes which may eventually lead to the approval of NUE12 for commercial cultivation by the farmers in the named countries.

### Sorghum

Sorghum [*Sorghum bicolor* (L.) Moench] is a tropical crop that is native to Africa and grown in several countries of the world. It is well-suited to semi-arid tropics because it is a hardy crop that can withstand harsh and water-scarce environments. According to FAOSTAT, (2020), sorghum production area in Africa is about 27.29 million ha, with total and average grain yield of 27.47 million metric tons and 1.01 ton/ha respectively. Nigeria and Ethiopia are the second and fourth largest producers of sorghum in the world, with United State occupying the top position (FAOSTAT, 2020). The grain is high in starch, which makes it a staple food in developing countries as well as feed for livestock and valuable feedstock for biofuel production in the developed countries. The crop’s comparatively small genome (around 818 MBp) has made it a model plant for researching the genetic components of drought resistance.

In Sub-Saharan Africa, one of the biggest problem of sorghum production is hemi-parasitic weed, belonging to the genus *Striga* (Muchira et al., 2021), It was estimated that *Striga* infestation negatively affect the livelihood of 300 million people in West Africa alone (Mboob, 1989). Although farmers often deployed cultural methods in managing *Striga*, however, genetic improvement of sorghum for resistance to *Striga* remains the most practical and effective solution. Since *Striga* is an obligated root parasite, its seeds only germinate when stimulated by chemical signal from the host plant (Rich and Ejeta, 2007). Therefore, low striga germination stimulant activity caused by genetic factors will be a good strategy for controlling yield losses due to *Striga* infestation (Pérez-Vich et al., 2013). Strigolactones—a group of compounds synthesised by most angiosperms as hormones to regulate branching of shoot and root (Gomez-Roldan et al., 2008; Rasmussen et al., 2012)—is the most potent germination stimulant among the sorghum root exudates (Gobena et al., 2017). A mutant allele at LGS1 (LOW GERMINATION STIMULANT 1) locus that drastically reduce *Striga* germination stimulant activity was identified by Gobena et al. (2017). This LGS1 is now a targeted gene for editing as Steven Runo from Kenyatta University, Kenya already used CRISPR-Cas9 system to develop a striga resistant variety of sorghum by knocking out *SgLGS1*. Similarly, mutagenesis of genes which encode Carotenoid Cleavage Dioxygenase—an enzyme involved in the biosynthesis of strigolactones in many plants species (Gao et al., 2018; Dutta et al., 2019)—was knock-out in sorghum by CRISPR system, resulting in significant reduction in *Striga* germination even though it negatively affected the yield (Hao et al., 2023).

### Millets

Millets are a diversified collection of small-seeded dryland cereals that are resilient to harsh climatic conditions, tolerant of poor soil conditions, and do not require excessive fertilizer or pesticide application. There are several types of millets, namely; foxtail millet (*Setaria italica*), pearl millets or bulrush millet [*Pennisetum glaucum* (L.) R. Br.], finger millet (*Eleusine coracana*), proso millet (*Panicum miliaceum*), kodo millet (*Paspalum scrobiculatum*), barnyard millet (*Echinochloa* spp.), browntop millet (*Panicum ramosum*) and little millet (*Panicum sumatrense*) (Upadhyaya et al., 2006). Climate change causes terminal moisture stress and erratic rainfall patterns at different stages of crop production across the world, thereby, reducing the yield of popular cereals like wheat and rice. However, millet due to its resilience to both biotic and abiotic stresses, has the potential to maintain a more stable yield with high quality. Hence, together with sorghum, these crops are critical for food security in the Sahel regions of West Africa, where they provide over 75% of the total caloric intake for highly food-insecure people in the region (Ndjeunga and Nelson, 1999).

Millets grains are frequently ground into flour and consumed in the form of porridge. They are also used for fermented drinks and other food purposes. When compared with other major cereal crops such as wheat and rice, millets are more nutritionally superior (Ragaee et al., 2006). The grains are high in various essential minerals-calcium, potassium, phosphorus magnesium, iron, and zinc-and vitamins, that help to reduce malnutrition (Hariprasanna et al., 2014; Elangovan et al., 2022). Also referred to as nutria-cereals or nutria-millets, millets are rich sources of essential fatty acids, proteins, carbohydrates, phytochemicals, and antioxidants. Millet grains are rich in polyphenols and particularly, finger millet starch has more amylose to amylopectin ratio, which confers several health benefits on the crop such as; reducing the risk of high blood pressure and heart diseases, and can help in the management of type 2 diabetes and obesity (Swaminaidu et al., 2015; Kumar et al., 2018).

Despite the innate ability of millets to cope with both biotic and abiotic stresses, the scourge of climate change is expected to be more biting on agricultural productivity in the future, leading to low yield and poor grain quality. Therefore, there is a need to further boost the resistance of millets against diseases caused by pathogens, combined stress caused by severe drought and excessive heat, and modification of plant architecture to prevent lodging and bio-fortification with essential micronutrients.

In Africa, pearl millet is the most cultivated, constituting 75% of annual millet production with the rest made up mostly by finger millet. However, unlike other cereals, pearl millet grains are particularly high in lipid content (Sharma et al., 2015). After milling of its whole grain, endogenous lipases are released and cause rapid onset of hydrolytic rancidity. This phenomenon makes the flours of most pearl millet varieties unstable 5–7 days after milling (Aher et al., 2022). Thus short shelf-life of pearl millet flour was identified as the major reason why pearl millet has remained unpopular (Goswami et al., 2020).

Generally, millets are regarded as orphan crops because previously, it has received less attention in terms of genetic studies and crop improvement from the scientific community. This is exacerbated by a lack of efficient transformation systems to successfully develop transgenic varieties or induce mutation in the genome through genome editing techniques because millets are recalcitrant to regeneration via tissue culture methods. In the last decade, millet has however gained attention because of its resilience to harsh climatic conditions and the supreme nutritive value of its grains. For example, the first draft genome of the pearl and finger millet was published by Hittalmani et al. (2017) and Varshney et al. (2017) (Ramu et al., 2023). The annotated genome of finger millets by Devos et al. (2023), is now available on Phytozome https://phytozome-next.jgi.doe.gov/info/Ecoracana_v1_1. It appears the efficiency of millet transformation is determined by factors like the choice of explants and the use of synthetic secondary metabolites like acetosyringone (Sood et al., 2019; Bhatt et al., 2021; Ceasar, 2022). It may also be influenced by specific millet genera being studied. For example, finger millet is predominantly transformed by *Agrobacterium*, with studies reporting the development of transgenic finger millet (Ceasar et al., 2011; Ignacimuthu and Ceasar, 2012; Satish et al., 2017). On the contrary, pearl millet seems to be transformed mainly by the biolistic method (Sood et al., 2019).

The first Genome editing in millet was reported by Lin et al. (2018) where the protoplast technology was used in creating CRISPR/Cas9 mutagenesis. Also demonstrated was the haploid induction in foxtail millet, by knocking out SiMTL gene using the CRISPR-Cas9 system (Cheng et al., 2021). More recently, Liang et al. (2022) also utilized *Agrobacterium*-mediated transformation to induce multi-genic mutation in the foxtail genome. A single-base editing in foxtail millet was also first reported in this study and it resulted in the creation of an herbicide-tolerant plant. Presently, it appears that not much success has been achieved in genome editing of millets, there is no report of genome editing of other millets apart from foxtail millet. Since pearl millet is widely cultivated in Africa, efforts must be intensified to address the problem of the short shelf-life of its flour. Recently, Aher et al. (2022) reported that the loss of functional triacylglycerol lipases in the grains of pearl millet is linked with low flour rancidity. This was demonstrated when a mutation in two genes *PgTAGLip1* and *PgTAGLip2* resulted in the loss of function and was consistent with low flour rancidity in some pearl millet varieties. Therefore, the two candidate genes will be good targets for genome editing to improve the shelf-life of pearl millet flour. Other genes which may be the target of genome editing for improvement of the resistance of millets to abiotic stresses such as drought have also been reviewed by Krishna et al. (2022).

### Maize

Maize (*Zea mays* L.), is considered the most important cereal crop in Central and Eastern Africa. For example, maize contributions to Kenyan economy include the generation of employment, serving as means of livelihood for many families as well as source of food security and foreign exchange earnings. Also, maize is the most important food crop in Kenya as ∼96% of the population consumed the crop as staple food every day (Njuguna et al., 2017). This is an indication that any significant yield loss due to abiotic or biotic stresses on the crop will have a damaging effect on food security in Kenya. An example of biotic stress that has caused significant yield loss in the country in recent time is Maize Lethal Necrosis (MLN), a viral disease that has caused between 23%–100% yield loss, estimated to be 180 million USD (Redinbaugh and Stewart, 2018). Conventional method for breeding high yielding maize varieties that are resistant to MLN is preferred over the use of pollution-prone method involving spraying of the MLN vector (Aphids and Thrips) (Awata et al., 2021). However, introgression of MLN resistant genes into susceptible varieties through backcrossing will take years to develop and may still suffer yield penalty as a result. To bypass this challenge, Kenya Agriculture and Livestock Research Organization in association with four other international partner organisations, embarked on genome editing project which seeks to identify and introduce MLN resistance genes into elite varieties (e.g., CML536) that are susceptible to the disease (CGIAR, 2022; Table 1). Since 2021, the MLN susceptible gene in elite varieties has been identified on maize chromosome 6 and has been edited to its resistance form against MLN disease. The role of the edited gene which now confers resistance on otherwise susceptible lines has been validated.

**TABLE 1:** Africa’s on-going and successful applications of genome editing for crop improvement.

Country	Project title	Challenges being addressed	Objective(s)	Target gene(s) and Phenotype(s)	Institution/affliation(s)	Lead scientist(s)
Nigeria^a^	Genome Editing for improved resistance to cassava Bacterial Blight (CBB) Disease	Yield and harvest loss due to CBB disease	To develop cassava resistant or with improved tolerance to CBB by disrupting gene(s) aiding disease establishment and spread	(T) MeSWEET10a gene, a susceptibility gene for CBB targeted with CRISPR-Cas9	National Root Crops Research Institute (NRCRI) Umudike	Dr. Ihuoma Chizaram Okwuonu
South Africa^a^	Genome editing of potato	Viral infection in Potatoes	To produce virus-resistant potatoes	(T) Eukaryotic initiation factor 4E (Eif4E)	Stellenbosch University	Prof. James R. Lloyd
South Africa^a^	High-throughput screening of genes associated with the response of cassava to geminivirus South African Cassava Mosaic virus (SACMV)	Yield loss due to susceptibility of African cassava varieties to cassava mosaic disease (CMD)	To silence genes putatively associated with the response to SACMV infection in susceptible and tolerant cassava landrace protoplasts using CRISPR gene editing 2. to identify the hub or key genes associated with SACMV tolerance in cassava protoplasts	Ubiquitin proteasome system genes (e.g. E3 ligases), transcription factor genes (e.g. WRKYs) and resistant genes (e.g. NLRs)	University of the Witwatersrand	Prof. Chrissie Rey
Uganda^a^	Application of targeted gene editing for development of high yielding, stress resistant and nutritious crops	Scarce information on molecular basics of flowering and lack of double	Production of fertile flowers and seeds by CRISPR-Cas9 mediated editing of endogeneous anti-flowering genes in cassava	(T). Phytoene desaturase and Terminal flower 1 (P). Photo bleaching and Early flowering	National Agricultural Research Organization (NARO), National Crops Resources Research Institute (NaCRRI)-Namulonge Campus	Dr. John Odipio
Ethiopia^a^	Improving oil qualities of Ethiopian mustard (Brassica carinata) through application of CRISPR/Cas 9-based genome editing	high level of erucic acid in elite varieties of Brassica carinata, beyond Nutritionally acceptable level	To develop B. carinata genotype with low erucic acid and glucosinolate for food and feed application To enhance the level of erucic acid for industrial application	(T). FAE1 and FAD2 genes for food GTR1 and GTR2 genes for feed and FAR and WS genes for Industries	Addis Ababa University	Prof. Teklehaimanot Haileselassie Teklu
Ethiopia^b^	Improving the susceptibility of Teff (*Eragrostis tef*) to lodging	Tef production is adversely affected by lodging	to develop semi-dwarf tef varieties	N/A	Ethiopian Institute of Agricultural Research (EIAR) and Donald Danforth Plant Science Center, USA	N/A
Kenya^c^	Modulation of energy homeostasis in maize to develop lines tolerant to drought, genotoxic and Oxidative stresses	Drought susceptibility in Maize	Metabolic engineering of Poly (ADP-ribosyl)ation pathway–a stress tolerance in plants by maintaining energy homeostasis during stress conditions	(T) Poly (ADP-ribose) polymerase (PARP1 and PARP2) (P) Maize tolerant to drought, DNA damage and oxidative stresses	Kenyatta University and Ghent University Belgium	Dr. Elizabeth Njuguna
Kenya^a^	Genetic improvement of banana for control of bacterial wilt disease	Control Xanthomonas wilt disease of banana in East Africa	To develop genome-edited banana resistant to bacterial wilt disease	(T) disease susceptibility ‘S’ genes (P) disease resistance	International Institute of Tropical Agriculture	Dr. Leena Tripathi
Kenya^d^	CRISPR/Cas9 editing of endogenous banana streak virus in the B genome of Musa spp. overcomes a major challenge in banana breeding	Difficulty in breeding plantain (AAB) due to existence of integrated endogenous banana streak virus (eBSV) in the B genome	Improve B genome germplasm and usefulness in breeding program	(T) (eBSV) (P) Prevent infectiousness of eBSV due to activation	International Institute of Tropical Agriculture and University of California, Davis, USA	Dr. Leena Tripathi
Kenya^a^	Gene editing to control maize lethal necrosis in Africa for improved maize productivity and grain harvests	Maize lethal necrosis (MLN) disease causes several losses to maize in Kenya and neighbouring countries	Introduce resistance against MLN disease directly into parent inbred lines of popular commercial maize varieties, which are currently susceptible to the disease, and reintroduce them to the farmer’s fields in Kenya with possibility of scaling out to other countries in East Africa	(T) A strong quantitative trait locus on maize chromosome 6 (P)high-level of resistance against MLN disease	Kenya Agriculture and Livestock Research Organization	James Kamau Karanja
Kenya^a^	Evaluation of Striga Resistance in Low germination Stimulant 1 (LGS1) mutant sorghum	Parasitic weed striga is a huge constraint to production of sorghum and other cereals crops	Evaluate LGS1 gene knockout in conferring Striga resistance in sorghum	(T) LGS1 (P) Mutant alleles at the LGS1 locus drastically reduce Striga germination stimulant activity	Kenyatta University	Prof. Steven Runo

N.B: sourced from:

^a^ Karembu and Ngure (2022).

^b^ Genome editing communication course in Ethiopia by AUDA-NEPAD (2024).

^c^ Njuguna et al. (2017).

^d^ Tripathi et al. (2019).

Drought is another factor that restricts maize output in Kenya in addition to other abiotic stress. This is because about 75% of the available land area in the country is arid and semi-arid (Zeila and Jama, 2005). Exposure of plants to drought stimulates overproduction of reactive oxygen species and increased oxidative stress which causes damage to primary metabolites (DNA, protein, Lipid proteins, and carbohydrates), cell death and loss of whole plant (Njuguna et al., 2017). The impact of drought on maize is phase specific, for example, when it occurs before anthesis, maize undergoes delay in flowering (Abrecht and Carberry, 1993), whereas if it is at grain filling stage, it can cause a more devastating effect like low grain yield (Schussler and Westgate, 1995). Recently, Njuguna et al. (2017) used CRISPR-Cas9 to knock down poly (ADP-ribose) polymerase (PARP), a gene that plays a major role in the maintenance of the energy homeostasis during stresses and significant increase in tolerance to oxidative stress resulted.

In 2016, there was an outbreak of fall armyworm (FAW; *Spodoptera frugiperda*) in Nigeria and São Tome, leaving large scale destruction in its trail (Goergen et al., 2016). This invasive insect has since spread to eastern African countries like Ethiopia, Kenya and Tanzania (Sisay et al., 2019). Spatial assessment of climate suitability of FAW indicated that Africa is generally favourable for this insect with the exception of Lesotho and South Africa (Senay et al., 2022). Moreover, most of the maize varieties are also susceptible to seasonal infestation of FAW. To control crop pests, three possible solutions exist; use of insecticides, integrated pest management (IPM) and genetic improvement of maize for resistance against FAW. Genetic solution appears to be the best option because maize production is largely done by smallholder farmers who cannot afford to spray their farm numerous times and IPM is not widely practiced in Africa (Alwang et al., 2019).

The TELA Maize Project led by Kenyan-based African Agricultural Technology Foundation, coordinated the insertion of a *Bacillus thuringiensis* gene which encodes Cry2Ab delta-endotoxin (Table 2), into the genome of drought tolerant maize varieties, thereby conferring insect protection and drought tolerance in TELA^®^ maize varieties through conventional breeding and biotechnology methods. Cry2Ab delta-endotoxin is a toxic protein which has lethal effects on the digestive system of lepidopteran and some dipteran insects (McNeil and Dean, 2011), however, it is not harmful to human and livestock, having been used in organic farming for over half a century to control insect pests. In a confined field trial of TELA^®^ maize in Nigeria by Oyekunle et al. (2023), it was reported that the transgenic TELA^®^ maize genotypes were resistant, not only to FAW but even to stem borer—another destructive pest of maize. Five TELA^®^ maize hybrids have been cultivated on a commercial scale by South African farmers since 2016 (Table 2). Also, Nigeria government, through the National Varieties Release Committee, in January 2024 approved the release of the seeds to of four TELA^®^ maize varieties (SAMMAZ 72T, SAMMAZ 73T, SAMMAZ 74T, and SAMMAZ 75T) to farmers and commercial production in the country (Alliance for Science, 2024). In Kenya, farmers awaits the release of three TELA^®^ maize hybrids (WE1259B, WE3205B and WE5206B) as it has been recommended by the Kenya Plant Health Inspectorate Service through National Performance Trial Committee (KALRO, 2024).

**TABLE 2:** Highlights of genetic engineering projects in various African countries.

Countries	Transgenic name	Gene/sequence introduced	Source	Gene product	Molecular function	References
Uganda, Kenya and Nigeria	VICRA Plus project	near full-length coat protein (CP) genes	cloned from CBSV and UCBSV	N/A	RNAi	Taylor et al. (2012)
		*NPTII*	*Escherichia coli*	neomycin phosphotransferase II enzyme	allows transformed plants to metabolize neomycin and kanamycin antibiotics during selection	Taylor et al. (2012)
		*IRT1* and *FER1*	*A.thaliana*	Iron transporter and Ferritin	Significantly increase the accumulation iron and zinc levels	Narayanan et al. (2019)
Kenya, South Africa, Nigeria	TELA Maize Project	cry2Ab2	*Bacillus* thuringiensis subsp. kumamotoensis	Cry2Ab delta-endotoxin	Confers resistance to lepidopteran insects	ISAAA, 2023b
		cry1A.105	*Bacillus* thuringiensis subsp. kumamotoensis	Cry1A.105 protein which comprises the Cry1Ab, Cry1F and Cry1Ac proteins	Confers resistance to lepidopteran insects	ISAAA, 2023b
		cspB	*Bacillus subtilis*	cold shock protein B	Maintains normal cellular functions under water stress conditions by preserving RNA stability and translation	ISAAA, 2023a
		nptII	*Escherichia coli* strain K12 Tn5 transposon	neomycin phosphotransferase II enzyme	Allows transformed plants to metabolize neomycin and kanamycin antibiotics during selection	ISAAA, 2023a
	Pod Borer-Resistant (PBR) Cowpeas	*Cry1Ab*	*Bacillus* thuringiensis subsp. *kurstaki*	Cry2Ab delta-endotoxin	Confers resistance to lepidopteran insects	AATF (2022)
		nptII	*Escherichia coli*	neomycin phosphotransferase II enzyme	allows transformed plants to metabolize neomycin and kanamycin antibiotics during selection	AATF (2022)
Nigeria, Uganda, Ghana	NEWEST Rice	*HvAlaAT*	*Hordeum vulgare*	alanine aminotransferase	Confers nitrogen efficiency on rice	Arcadia Biosciences (2018)
Nigeria, Kenya, Burkina faso South Africa, Egypt	Africa Biofortified Sorghum project	*Hv-HGGT*	*Hordeum vulgare*	homogentisate geranylgeranyl transferase	Increase Vitamin E	AHBFI (2023)
		*At-DXS*	*Arabidopsis thaliana*	1-deoxy-D-xylulose-5-phosphate synthase	Increase carotenoids	AHBFI (2023)
		*Zm-PSY1*	*Zea mays*	Maize phytoene synthase 1	Increase carotenoids	AHBFI (2023)
		*CRT I*	Bacterial (*Erwinia*)		Increase Vitamin A biosynthesis	AHBFI (2023)
		*PMI*	*Escherichia coli*	phosphomannose isomerase	A selection gene instead of antibiotic markers	AHBFI (2023)

### Cassava

Cassava is a major crop in the tropical and subtropical regions of the world, cultivated for its starch-rich swollen roots (Okogbenin et al., 2002; Okogbenin et al., 2003; Okogbenin et al., 2012). Agronomical qualities like resilience against stress-prone environments, adaptability to subsistence farming systems and high starch content of its storage roots, makes it the crop of choice for millions of smallholder farmers (Montagnac et al., 2009; Howler et al., 2013). Harvested storage roots are processed and converted to various food products in sub-Sahara Africa (SSA), providing over 50% of the caloric intake of one-third of the entire population (Okogbenin et al., 2007; Ewa, 2021). Nigeria is the world’s leading producer of cassava (Table 3). However, the country is still not at its full potential in terms of yield (tonnes/hectare), as it produces <80% of the world average (FAOSTAT, 2017). In fact, over 20% decline in yield/hectare of storage root was reported between 2007 and 2017, despite a significant increase in the area of land cultivated within this period (Otekunrin and Sawicka, 2019). Factors, such as susceptibility to diseases and high post-harvest loss due to rapid physiological deterioration are major limitations preventing Nigeria from reaching its full potential in cassava production (Fregene et al., 1997; Fregene et al., 2000; Akano et al., 2002; Ogbe et al., 2006; Akinbo et al., 2007; Akinbo et al., 2011; Akinbo et al., 2012a; McCallum et al., 2017; Zainuddin et al., 2018). However, with biotechnology approach, scientists from national agricultural research institutions in Brazil, Nigeria, Ghana and Uganda, are rapidly building resistance to green mite, whitefly, cassava mosaic disease and post-harvest physiological deterioration (PPD) with the support of their counterparts at the International Center for Tropical Agriculture (CIAT) in Colombia (Fregene et al., 1997; Akano et al., 2002; Rudi et al., 2010; Akinbo et al., 2012b).

**TABLE 3:** The total land area, production of major crops in Nigeria and export quantity.

Crop	Land area (×10^3^ ha)	Total production (×10^3^ tons)	% In world production	Export quantity (tons)
Cassava, fresh	9,085.74	63,031.38	20.02	3.6
Yams	5,902.18	50,377.34	67.04	194.13
Taro	816.098	3,216.12	25.9	0.01
Sesame, Seed	496.71	440	6.9	138,333.33
Sweet Potato	1,510.41	3,943.05	4.44	83.96

Other factors that contribute to final yield of cassava which are currently being worked on for improvement include storage root yield and starch content (Okogbenin et al., 2003). Significant progress has been made in this area since Cassava Source-Sink (CASS) project began. Funded by Bill and Melinda gate foundation, CASS project focuses on combining techniques in plant biotechnology with physiological processes to develop cassava genotypes with increased storage root and starch yield. The goal of the project is to boost the income of smallholder farmers in SSA (Sonnewald et al., 2020).

Due to heterozygosity of the crop, simultaneous stacking of multiple (desirable) genes in a cassava variety through plant breeding methods is very challenging (Fregene et al., 2006; Beyene et al., 2018). However, biotechnological approach has been utilized to overcome this (Fregene et al., 1997). The technique is considered as the most reliable option for combining multiple traits in a crop. For instance, CASS project adopted systematic gene stacking approach, involving simultaneous incorporation of foreign genes for increased storage root and starch yield (Sonnewald et al., 2020). To this effect, several transgenic lines are currently under confined field trial (CFT) in National Chung Hsing University, Taiwan and International Institute of Tropical Agriculture Ibadan Nigeria (Sonnewald et al., 2020; CASS, 2023).

Although, cassava is regarded as a food security crop, meals made from its swollen storage roots are considered unwholesome, due to the absence of essential minerals such as iron and zinc in the crop (Sayre et al., 2011). Deficiency of zinc and iron is common among children under the age of five and pregnant women in Nigeria (WHO, 2008; NMHN 2005; Table 3). Genetic engineering method has also been utilized in the biofortification of the crop by co-expression of iron transporter and ferritin genes from *A. thaliana* which results in accumulation of iron and zinc in the storage root, up to a substantial level in human diet (Narayanan et al., 2019). Similarly, a transgenic cassava variety developed by Beyene et al. (2018) not only shows high pro-vitamin A in its storage roots but also exhibited longer shelf life when compared with ordinary variety. Taylor et al., 2004; 2012; Siritunga and Sayre 2003; Zhang et al., 2005 similarly worked on other traits to improve cassava qualities.

Since genome editing of Cassava was first reported by Odipio et al. (2017) using CRISPR/Cas9 system, the technique have been effectively utilized to control high accumulation of toxic compounds in the leaves or roots of cassava. For example, Juma et al. (2022), successfully lowered cyanide levels in cassava by inducing mutagenesis in *CYP79D1* gene—encodes Valine N-monooxygenase 2 which catalyses major reaction in the synthesis of cyanogen in cassava—using CRISPR/Cas9 system. Similarly, other traits such as herbicide tolerance, modification of root starch structure and disease resistance have been possible through CRISPR/Cas9-mediated targeted mutagenesis (Bull et al., 2018; Hummel et al., 2018; Gomez et al., 2019; Veley et al., 2021).

### Yam

White yam (*Dioscorea rotundata*) is the most popular species among tuber-bearing crops in the family Dioscoreaceae. According to FAOSTAT 2021, Nigeria was the largest producer of this crop in the world. Its economic value is derived mainly from the sale of its starch-rich tuber and day-to-day consumption, providing about 285 dietary calories for 300 million people in Sub-Saharan Africa (Adejumo et al., 2013). Apart from its rich starch deposit, yam tuber also contains higher a vitamin C and crude protein content than the swollen storage roots of cassava, making it a healthier food source (Baah et al., 2009). It is also an important source of secondary metabolites such as steroidal saponins (diosgenin), diterpenoids, and alkaloids, which are utilized in the manufacturing of pharmaceutical products (Mignouna et al., 2008; de Lourdes Contreras-Pacheco et al., 2013).

A major constraint to yam production is infestation with parasitic nematodes, insects, fungi, and viruses, which reduce both the yield and quality of tubers (Asante et al., 2008; Asala et al., 2012; Zaknayiba and Tanko, 2013). Amusa et al. (2003) claimed that nematode infestations might also result in a sizeable yield loss. Despite Aighewi et al. (2015) reporting that viruses can cause a 50% yield loss. These problems have become more severe lately due to the repetitive usage of pest-infested tubers, usually from previous cropping seasons for planting with each successive year (Amusa et al., 2003; Aidoo et al., 2011). Inter-state exchange of these infected planting material further worsens the situation, as mixed viral infections were detected in a survey carried out in Guninea-Savana. Zone of Nigeria where yam is mostly produced (Asala et al., 2012). As such, Aighewi et al. (2015) recommended the use of planting material developed by tissue and organ culture as a better alternative to the pervasive traditional way of yam propagation, stating that it is fast and guarantees the production of disease-free planting material. Also, yam clones can be cleaned from virus disease by cryotherapy, a method which involves short-term treatment of the infected samples in liquid nitrogen to eradicate the pathogens (Wang and Valkonen, 2009). Eradication of bacteria, viroids, and viruses using this method have been reported in the planting material of economically important crops such as banana (Helliot et al., 2002), potato (Wang et al., 2006) and sweet potato (Wang and Valkonen, 2008).

Transfer of useful genes from wild families of yam to elite varieties has proven difficult (Spillane and Gepts, 2001) because yam improvement through conventional breeding methods is constrained by poor flowering, polyploidy, and heterozygosity (Mignouna et al., 2008). However, a protocol for *Agrobacterium*-mediated transformation of yam has been developed (Nyaboga et al., 2014), and this has opened a new opportunity for developing yam varieties that are resistant to viral and nematode infection through genetic engineering, although optimization of this protocol for different yam genotypes may be necessary for effective and increased transformation efficiency. Additionally, knowledge of nematode-host interactions could be explored through a biotechnological approach to target yam-nematode activity by disrupting the nematode’s infective stage which may reduce passage potency through yam tissues, fecundity, establishment in yam cells and/or feeding ability on susceptible yam cultivars. In a study by Jaouannet et al. (2013), repression of calreticulin gene expression in *Meloidogyne incognita* by RNAi–RNA interference led to a decrease in the ability of the nematode to infect *Arabidopsis thaliana* and overexpression of *M. incognita* calreticulin gene in *A. thaliana* increased the susceptibility of the plant to the nematode infection. The study clearly shows the role that biotechnology can play in manipulating host-pathogen interactions. Fosu-Nyarko and Jones, (2015) suggested that RNAi technology possesses great potential in conferring resistance to several nematode species on the plant, unlike the introgression of natural resistant genes. Henceforth, genome editing may also play a key role in improving various traits in yam. The first successful CRISR/Cas9-based genome editing and validation in yam was reported by Syombua et al. (2021). *Agrobacterium* harbouring the CRISR/Cas9 vector was used in targeted mutagenesis of the phytoene desaturase gene in *D. rotundata*.

### Cocoyam

Cocoyam is often used interchangeably in literature for species of the two most cultivated genera, *Colocasia* and *Xanthosoma*, belonging to the family Araceae. The genus *Colocasia* spp. has between 11 and 16 species (Long and Liu, 2001; CABI, 2013), whereas *Xanthosoma* spp. has about 60 species (Bown, 2000; Stevens, 2012). Taro, or old cocoyam (*C. esculenta*) and tannia or new cocoyam (*Xanthosoma sagittifolium*) are by far the most cultivated species in the world (Ubalua et al., 2016; Degefa and Anbessa, 2017). In West Africa, *X. sagittifolium* is the main edible aroid and has overtaken *Colocasia esculenta*, probably because its plant is more robust and tolerant to drought and the cormel, a rich source of starch for domestic and industrial use, is adapted to preparing indigenous foods like fufu (Onokpise et al., 1999; Jennings, 2009). The small granule size of its starch (taro) is easily digestible, thereby making it a healthy energy food for children (Adekiya and Agbede, 2016). Nigeria is the largest producer of cocoyam in the world (FAOSTAT, 2021) and approximately 86.27 × 10^6^ million hectares of arable land are still available to further scale-up the production of the crop in the country (Chukwu, 2015). Despite the enormous potential of cocoyam production in the country, production is limited by a lot of factors, among which, frequent incidence of pests and diseases is a major challenge. In 2009, cocoyam production was nearly disrupted completely because of the severity and pervasiveness of taro leaf blight which attacked farms in Nigeria (Ubalua et al., 2016). Although chemical control of pest is considered an option in the control of diseases, an increase in the prices of pesticides, destruction of the underground cocoyam corm-setts by broad-spectrum herbicides in the farm and the toxicity of some active ingredients in the pesticides have discouraged many farmers from subscribing to this option (Acheampong et al., 2015).

To control pest infestations, developing genetically resistant cultivars is recommended as the safest and most economical option. Improvement by conventional breeding methods is difficult because cocoyam rarely flowers naturally but this can be induced artificially (Tambong and Meboka, 1994; Jennings, 2009). Techniques for pollen storage and germination have been developed to facilitate sexual crossing in the crop (Agueguia and Fatokun, 1987). However, some of the methods for artificial flower inducement may be cultivar-dependent as observed in the cocoyam cultivar “Bun Long” which does not respond to inducement by gibberellic acid (Ivancic et al., 2004). Tissue culture has been deployed in the multiplication of cocoyam cultivars that are resistant to taro leaf blight through collaborative research by the NRCRI and the European Union since 2010. This effort is laudable, as it encourages the development and distribution of resistant cultivar/s among farmers. Traditional cocoyam breeding methods can take up to 10 years, whereas genetic engineering can be used as a substitute technique to produce varieties with highly desirable traits. The Glucuronidase (*Gus*) gene (Fukino et al., 2000) and rice chitinase gene *chi11* (He et al., 2010) have been transformed into cocoyam by particle bombardment, although, transformation efficiency was low. *Agrobacterium*-mediated transformation of cocoyam (Bun long Cv.) with rice chitinase gene *chi11 and* wheat oxalate oxidase gene *gf2.8* led to the production of a transgenic potato that is resistant to *Sclerotium rolfsii* and *Phytophthora colocasiae* (He et al., 2008, 2013). This method appeared to be more promising, because its transformation efficiency is high (between 1% and 3%), opening up a greater opportunity for other crops than what was obtained for the particle bombardment method (<0.5%). The protocol for *Agrobacterium-*mediated transformation of Taro has been developed by He et al. (2015).

### Groundnut

Groundnut, also known as peanut, is a leguminous crop cultivated in the semi-arid and subtropical regions of the world for its oil-rich seed and consumed as food and feed. Groundnut seed which contains 44%–56% oil is very high in unsaturated fatty acid 85% (Sabate, 2003), making it a top choice among oilseed crops. The protein content of the seed is about 22%–30%, also it is a rich source of essential minerals and vitamins. Groundnuts, with their high seed oil and protein contents, play a crucial role in preventing malnutrition and guaranteeing food security. Frequent nut consumption is associated with lower rates of coronary artery disease. Also, nut-rich diets improve the serum lipid profile of participants in dietary intervention trials. Despite their high-calorie density, groundnuts produce satiety, limited energy absorption, and enhanced energy expenditure after eating, hence they do not significantly contribute to weight gain (Sabaté, 2003; Mattes et al., 2008).

Groundnut haulms, a by-product is not only gaining prominence as a fodder source for feeding livestock during the dry season when green grasses are unavailable (Ajeigbe et al., 2014) but also due to dwindling arable land and water resources occasioned by climate change in the Sahel region of West Africa (Blümmel et al., 2012). Nigeria ranks fourth in the world and first in Africa in groundnut production, contributing about 2.42 million MT out of the 45.3 million MT produced in 2017 (FAOSTAT 2017). Regardless of Nigerian’s ranking globally in groundnut production, the country seems not to be making progress in the production of this crop, for example in 2017, the USA surpassed Nigeria in groundnut production for the first time in over 10 years with a production of 3.28 million MT (FAOSTAT 2017). This may not be unconnected with Africa’s poor groundnut yield/ha, reported to be (929 kg/ha) unlike those obtainable in Asia and America (2,217 kg/ha) and (3,632 kg/ha) respectively (FAOSTAT, 2014).

Additionally, Ajeigbe et al. (2014), identified biotic and abiotic stresses as major factors constraining groundnut production in Nigeria. For example, groundnut is highly susceptible to aflatoxin contamination, which are secondary metabolites synthesised by aflatoxigenic fungi like *Aspergillus flavus* and *A. parasiticus* after infecting the pods or seeds at the preharvest and post-harvest stages. According to Wilson and Stansell (1983) and Cole et al. (1995), aflatoxin contamination is an extremely variable trait that arises largely under heat and drought stress, and the location where groundnut is primarily grown in Nigeria is especially prone to these abiotic stresses. Aflatoxins are highly toxic to humans and have been linked to liver cancer, suppression of the immune system, and retarded growth in children (Bhatnagar-Mathur et al., 2015). Several countries throughout the world have been obliged to implement rigorous guidelines for permitted levels of aflatoxins in groundnut imports. As a result, Africa loses around USD 500 million per year in export trade due to systematic rejections of export crops and animal products with unacceptable levels of aflatoxins (Janila and Nigam, 2013).

Competitive atoxigenic fungal technology and deployment of promiscuous atoxigenic *Aspergillus* are some of the preharvest strategies that have been effectively used to reduce the level of aflatoxin contamination but it poses the problem of compromising the quality of the kernels and hygiene. Thus, the development of varieties that are resistant to the preharvest infestation of groundnut by *A. flavus* remains a viable option, though it has remained a challenge for peanut breeding programs (Janila and Nigam, 2013; Bhatnagar-Mathur et al., 2015). To effectively minimize the occurrence of pre-harvest aflatoxin contamination, the mechanism initially proposed by Holbrook et al., 2009 and corroborated by Janila et al. (2013) involves the identification of groundnut genotypes that are resistant to either drought or root-not nematode (Holbrook et al., 2009). This was because significant positive correlations were observed between resistance to these stresses and aflatoxin contamination. Subsequently, the potential for enhancing antifungal activities in groundnut seeds using marker-assisted selection was demonstrated by Yu et al. (2020), where SNP marker system was used to identify gene markers in two novel groundnut genotypes linked to genes that confer resistance against aflatoxin contamination.

Various strategies have recently been deployed for the transformation and development of transgenic groundnuts with alteration in the complex interaction between pathogen and groundnut-host system. This involves the use of genes that encode proteins/enzymes (antimicrobial peptides like defensins) which activate defence mechanisms against fungi and aflatoxin or host-induced silencing of Aspergillus genes encoding key enzymes involved in fungal sporulation or aflatoxin production. Overexpression of *Medicago sativa* Defensin 1 and *Medicago truncatula* Defensing 4. 2 and through HIGS of the aspergillus gene; *AFlM* (Ver-1)–encodes versicolorin dehydrogenase–and *AflP* (omtA)–encodes methyltransferase–resulted in high level of resistance in groundnut to aflatoxin production (Sharma et al., 2018). Similarly, multiplexed HIGS of *A. flavus* genes (*AflM*, *AflR*, *veA* and *nsdC*) also enhance resistance to infection caused by Aspergillus and aflatoxin contamination (Prasad et al., 2023).

With the successes recorded so far through various strategies mentioned, the future of genome-edited groundnut with high resistance to aflatoxin is very promising. Firstly, there is abundant information on the genomic information (whole genome sequences and annotations) of *A. flavus* to study its biology (Payne et al., 2006; Payne et al., 2008; Nierman et al., 2015; Ohkura et al., 2018; Fountain et al., 2020; Bharose et al., 2024). Also, the reference genome of groundnut is available (Zhuang et al., 2019). The first CRISPR-based editing of *Fad2*-the gene encodes the enzyme that catalysis the conversion of oleic acid to linoleic acid (Schwartzbeck et al., 2001)- in groundnut was reported by Yuan et al. (2019) and it resulted in elevated levels of oleic acid and reduction in linoleic acid for improved oil quality and better health benefits. Neelakandan et al. (2022) went further to create the first induced base editing of FAD2 genes in groundnut, using CRISPR/Cas9.

### Sesame

Sesame (*Sesamum indicum* L.) is among the ancient oil-yielding crops. Its seeds, when decorticated, bear one of the highest oil contents. The oil is made up of 83%–90% unsaturated fatty acids (Fukuda et al., 1985; Anilakumar et al., 2010). The seed is also rich source of protein, vitamins, minerals and lignans (methylenedioxyphenyl compounds like sesamolin, sesamin, sesamol and tocopherols (Fukuda et al., 1985). In 2021, Nigeria was the second largest producer of sesame in Africa and the sixth in the world. In terms of export quantity, the crop was only second to cocoa, suggesting that sesame has high potential for contributing to the country’s foreign earnings (FAOSTAT, 2021). According to Tukura and Ashindo (2019) Nigeria earned 139 million and 1.4 billion USD from exporting sesame in 2010 and 2012 respectively. However, a review of sesame seed production in Nigeria from 2003 to 2012 by Umar et al. (2014) revealed that increase in production experienced within this period was due to increase in land area used for cultivation rather than an increase in average yield per hectare, which was very low and mostly similar to the world’s average of 0.49 t/ha (FAO, 2012). Early senescence and extreme susceptibility to biotic stresses like bacterial blight (*Xanthomonas campestris* pv. sesame) and powdery mildew (*Oidium erysiphoides*), and abiotic stresses like photosensitivity and waterlogging are major constraints to increasing sesame yield (Dossa et al., 2017). Even though studies have shown that the wild species of the crop is a repository of desirable genes that can help elite varieties cope with these stresses (Kolte, 1985; Brar and Ahuja, 2008), post-fertilization barriers remain a major hindrance to transferring desirable genes into elite varieties by conventional breeding approach (Tiwari et al*.,* 2011). Thus, genetic engineering is the only available option for the transfer of those useful genes from the wild species into the elite varieties. Since Yadav et al. (2010) reported the first successful *Agrobacterium*-mediated transformations of sesame, other authors, like Al-Shafeay et al. (2011) and Chowdhury et al. (2014) have also achieved similar results in about 42.66% transformation efficiency. *Agrobacterium*-mediated transformation of sesame brought about the development of multiple-stress tolerance in the crop by overexpression of Osmotin-like proteins (*SindOLP*) gene (Chowdhury et al., 2017). Morphological features of the plant, like the number of capsules per plant, the number of grains per capsule, grain weight, plant height, length of capsules, number of capsules per axil and axis height of the first capsule has been associated with grain yield of sesame (Dossa et al., 2017; Teklu et al., 2022). Wei et al. (2015, 2016) stated that problems associated with low yield in sesame production may be solved through functional genomic study involving multigenic assemblage of experimentally determined genes such as *SiGA20ox1* and two candidate genes for plant height (*SiDFL1*) and (*SiILR1*) in a transgenic sesame line delivered by *Agrobacterium*-mediate transformation.

### Sweet potato

*Ipomoea batatas*, the common sweet potato, is an important staple food of many tropical and temperate countries, as it ranks fifth in developing nations in terms of economic value and seventh for energy consumption (Loebenstein, 2009). It plays an important role in nutritional improvement, as well as serving as raw materials in the processing of feeds, starches and bioethanol in various industries (Antonio et al., 2011). Africa is the second largest producing region, with almost 17% of the world’s production and more than 42% of the world’s area, mainly for human consumption (Zhang et al., 2018). Globally, traditional breeding has significantly contributed to trait improvement in the crop (Thottappilly and Loebenstein, 2009). Crop variety is the main variable often manipulated by farmers to raise yields. Egeonu and Akoroda, (2010) characterized and evaluated for sequential selection, 125 clones of sweet potato for different end-uses. In recent times, the biofortification of sweet potato with provitamin A carotenoids have proven to be an economical and potentially sustainable strategy to alleviate vitamin A deficiency (VAD) in developing countries (Tumuhimbise et al., 2013). The flesh of sweet potatoes can be white, yellow, purple, or orange in colour. Based on this diversity, it has been linked to acceptance in terms of nutrition and taste. Particularly, orange-fleshed sweet potato (OFSP) types are the most affordable and year-round source of vitamin A available for low-income families (Nkhata et al., 2020). Initially, most orange-fleshed sweet potatoes had lower dry matter content and poor environmental adaptability than ordinary white sweet potato varieties (Mwanga and Ssemakula, 2011). However, several years of breeding has produced OFSP with improved yield, flavor, drought resistance, dry matter content and early maturation, resulting in increased adoption by farmers (Hummel et al., 2018; Jenkins et al., 2018). National Root Crops Research Institute (NRCRI) has come up with the development of 2-orange and 1- white-fleshed sweet potato to improve the nutritional wellbeing of Nigerians. Additionally, purple-fleshed sweet potato clones, capable of accumulating anthocyanin (powerful antioxidant) in their storage roots have been developed (Montilla et al., 2011). Researchers have reported the encouraging health benefits of OFSP intervention into the staple food, currently available in more than three African countries, including Nigeria. Neela and Fanta, (2019) reviewed the detailed nutritional composition (proximate, mineral, carotenoids, vitamins, phenolic and antioxidant property’s role in Vitamin A deficiency (VAD) management and different food products that can be made from OFSP. Scientists at IITA in Nigeria and in Ethiopia have also developed methods to produce virus-free sweet potato plant through meristem culture (IITA, 1989; Dugassa and Feyissa, 2011). Nonetheless, a lot can still be done on sweet potato production. Biotechnological tools, such as gene transfer would be very effective in its improvement, as they will enable direct introduction of desirable genes from pre-adapted cultivars. In addition, selection using DNA markers would accelerate conventional breeding programmes in Nigeria. The whole genome sequence of sweetpotato is yet to be available publicly and this has limited research effort directed at improving its agronomic traits. However, the genome sequence of its two diploid relatives I. triloba and I. trifida has been done and it can still be utilized as reference genome for studying hexaploid sweetpotato (Wu et al., 2018). Targeted mutagenesis of genes encoding key enzymes in starch biosynthetic pathway (GBSSI and SBEII) was the first report of genome editing in sweetpotato (Wang et al., 2019).

### Tomato

Globally, Tomato is a vegetable crop, preceded only by potato in terms of production and global consumption (Dias, 2012). It is an important industrial and cash crop in many countries, because of the economically attractive and rich nutrient composition of the fruit, with the attendant health-associated benefits (Willcox et al., 2003). Tomato is being used in Nigeria as ingredients of meals, salads, ketchup, soups and sauces from time immemorial (Akaeze and Aduramigba-Modupe, 2017). Nigeria ranks 11th among the largest tomato producing countries in the world, with a production of 4.1 MT (FAOSTAT, 2017). However post-harvest loss of tomato is very alarming, estimated at 30%–50% in the country (Ajagbe et al., 2014). This is largely because the shelf life of the crop is particularly shortened (48 h) in the tropics (Muhammad et al., 2011), coupled with poor post-harvest handling and storage. To meet domestic demand for tomato, Nigeria imports 150000 MT of tomato paste annually, valued at 170 million USD (Ayedun and Akande, 2023). Moreover, a survey by Ugonna et al. (2015) revealed that only 20% of processed tomato are produced in Nigeria while the remaining 80% is imported. The fruits of Flavr-Savr™, a transgenic tomato variety released in 1994, was the first commercially available food crop. Since then, many other GM tomato varieties have been commercialized. Flavr-Savr™ was developed by inhibition of polygalacturonase enzyme, responsible for pectin molecule degradation in the cell wall, thereby causing delayed softening of fruit and elongated shelf life (Baranski et al., 2019). RNAi silencing and CRISPR-based mutation of ripening-related gene which encodes pectate lyase, caused the fruits to be firmer over a long period (Yang et al., 2017; Wang et al., 2019). Decrease in the activities of enzymes (polygalacturonase, tomato β-galactosidase, cellulase β-D-xylosidase) involved in cell wall modification as result of RNA silencing of *SIFSR* gene also elongated shelf-life significantly (Zhang et al., 2018). This makes *SIFSR* gene a modification a potential target for improving potato shelf-life. Similarly, overexpression of certain genes like *SICOBRA-like* gene, by genetic engineering revealed their role in elongating tomato shelf life (Cao et al., 2012).

The susceptibility of the current tomato cultivars to diseases and pests is another barrier to tomato production (Ugonna et al., 2015). Although, certain cultivars including H9-1-6 and Ronita are either resistant to leaf diseases or moderately resistant to root-knot nematode, they are susceptible to a host of others. The genetic basis of tomato is progressively becoming narrower from the time of domestication in its centres of origin to its spread to other parts of the world because selection was solely aimed at increasing yield (Gruber, 2017). Miller and Tanksley, (1990) reported that the genetic variation existing between tomato cultivars is <5% while the rest is embedded in the wild species of the genus. Some of the wild species that show high degree of homosequentiality in their chromosomes have been successfully exploited in tomato breeding for improving traits such as tolerance to adverse weather conditions, quality of fruit, pathogen and insect resistance (Zhang et al., 2002; Rodríguez et al., 2006; Bai and Lindhout, 2007). Effective use of wild species of tomato for improving elite varieties requires accurate understanding of the genetic factors responsible for desirable agronomic traits in this wild species. However, most breeding projects are conventional in design, which makes simultaneous studying of multiple traits a difficult task. Since domestication of tomato from the wild species was almost solely driven by yield while other important traits such as resistance to diseases and tolerance to stress largely remained with the wild species (Kik et al., 2010), genome editing already proved useful in domesticating wild tomato (Zsögön et al., 2018). Using CRISPR-system technology, Zsögön et al. (2018), edited six loci—associated with high yield in elite tomato cultivars—in *Solanum pimpinellifolium* which resulted in alteration in the plant morphology alongside fruits size, number and higher accumulation of antioxidant lycopene.

### Molecular farming

In 2021, Nigeria joined the rest of the world in the effort to develop an effective vaccine against the deadly coronavirus. Consequently, the Nigeria Vaccine Policy (NVP) was established for the first time in order to promote domestic vaccine manufacturing and guarantee autonomy in vaccine accessibility. A possible area of research that ought to be looked into is the use of plants in the production of effective vaccines against both communicable and non-communicable diseases. This is because the government offered to support and fund vaccine research and development as part of the implementation strategies to achieve the aim and objectives of the NVP. For over three decades, plants have been used as a bio-factory to manufacture pharmaceutically important recombinant proteins (such as plasma proteins, antibodies and cytokines) and diagnostic reagents through molecular farming (Fischer and Buyel, 2020). More recently, utilization of plants as subunit vaccine is gaining prominence, because it is a quicker and safer alternative to conventional vaccine development which is based on attenuation or inactivation of specific virus (Capell et al., 2020). The recombinant protein is produced in plant using deconstructed vector mediated by agroinfiltration with *Agrobacterium tumefaciens* (Gleba et al., 2014). Before the advent of molecular farming, there are established platforms such as microbes (e.g., *Escherichia coli*) and various mammalian cells cultures used in the industrial manufacturing of biologics. Plant is yet to displace these major platforms because investment in the industry and the existing regulatory framework favours these earlier established platforms. However, since Nigeria is just developing capacity in this area, government can take advantage of plants as the platform for the manufacturing of important biologics. This is because, with plants as the platform, the production of biologics can be done on a massive level and scaled up rapidly to cater for unexpected surge in demand and do not support growth of human pathogens (Ma et al., 2003; Whaley et al., 2011).

## Origin of anti-GMO campaign in the world

In the early 1980s, scientists established *Agrobacterium*-mediated transformation in plants and identified CaMV 35S promoter, which can facilitate gene expression (Odell et al., 1985; Herrera-Estrella et al., 1992). These two important findings were later combined to engineer the production of the first transgenic herbicide tolerant plant (Shah et al., 1986). The three milestones led to the launch of plant biotechnology on a grand scale and created an avenue for the development of the field of recombinant DNA technology. Crops produced through this “unnatural method” of altering plant genetic material are regarded as genetically modified GM-crops (Abdul Aziz et al., 2022), although in the true sense, all crops, with respect to their current genetic make-up, originated from long time-controlled breeding, selection and domestication processes, which have genetically modified them from their wild state. However, regardless of the huge potential of GM-crops and their new possibilities in ensuring food security, there is a global skepticism about the consumption of such crops, because they are perceived to portend great risk to human health. For instance, heated debates among scientists on the safety of GM-crops came about from the findings of Arpad Pusztai, a protein scientist, who tested the effect of consumption of transgenic potato on rats, and afterwards, opined that consumption of such potato by the rats was the reason for the lack of good health of the animals (Enserink, 1998; 1999a). Even though no firm conclusion was made in his research to affirm the risk of GM-crops to human health, his work stared up anti-GM campaigns and fuelled further research. In the publications by (Ho et al. (1999) and the Institute for Agriculture and Trade Policy (1999) **,** CaMV 35S promoter was regarded as the culprit in the supposed unwholesome effect of GM-crop, because it was assumed to be virus, whereas it is just a short stretch of DNA (Amack and Antunes, 2020). However, the concern raised by the Pusztai data was later debunked, after his data was re-examined by external experts and The Royal British Society, who found the framework of his experiment inconsistent and thereafter concluded that any finding from his work should be discarded as it lacked merit (Enserink, 1999b; Kuiper et al., 1999; The Royal Society, 1999; Yang, 2005). However in Nigeria, pressure groups made up of the coalition of civil society groups, farmers, students and faith-based organisations have constantly protested the adoption of GM-crops (Omeje, 2019). The argument against GM-crops is that it poses great threat in the areas of toxicology, allergy and immune dysfunction. These claims mostly originated from (Fagan et al., 2015), who stated that the process of genetic engineering could disrupt pristine proteins or metabolic pathways, which may result in the production of toxins or allergens in food. Predictive animal testing is a method that is commonly used to assess food allergies, both for genetically modified and non-GM foods. However, it has been found to be inadequate. (Wal, 2015). Till date, despite efforts toward developing animal models for accurate prediction of sensitivity to allergy, none has proven to be predictive (Ladics and Selgrade, 2009; Goodman, 2015; Kazemi et al., 2023). Consequent on this fact, the National Academies of Sciences, Engineering, and Medicine (2016), suggested the use of pre-commercialization tests to make rough predictions on relationships between consumption of GM-crops and the prevalence of some human diseases.

## Plant biotechnology protest in Nigeria: the case study of transgenic cassava trial

Nigeria is yet to reach its full potential in cassava production despite being the leading producer. The main factor responsible for this is post-harvest loss due to rapid physiological deterioration of its swollen storage roots that are rich in starch. This reduces the post-harvest value to about 40% lower than its original worth and impact farmers’ income negatively. Addressing this problem will positively impact Nigeria in food security and boost socio-economic status of many farmers. Using techniques in plant biotechnology, the lab of Prof. Samuel C. Zeeman developed transgenic cassava called AMY3 RNAi transgenic lines. It is so named because the transgenic line lacks the activity of one of alpha-amylase isoforms AMY3, an enzyme involved in starch degradation in its storage roots and the named lines showed prospect of slowing down post-harvest deterioration of cassava storage root. In 2017, application for confined field trial of AMY3 RNAi transgenic lines at International Institute of Tropical Agriculture was approved by NBMA. Going forward, objections were raised against this, stating that the concerned lab has worked mostly on Arabidopsis previously (Fulton et al., 2008; Liu et al., 2023). However, it is a known fact that the evolution of starch metabolism is primarily conserved across angiosperm, which implies that knowledge gained from studying starch metabolism in the model plant, is mostly applicable to other plant species (Pfister and Zeeman, 2016). Although slight differences have been reported on biosynthesis and degradation of starch in storage organs like tuber and endosperm, Prof. Zeeman’s lab has demonstrated profound knowledge on starch metabolism in crops like cassava (Zhou et al., 2017; Bull et al., 2018; Wang et al., 2018) and potato (Hussain et al., 2003; Ferreira et al., 2017; Samodien et al., 2018) with several published reviews on this subject (Smith et al., 2005; Zeeman et al., 2010; Santelia and Zeeman, 2011; Stitt and Zeeman, 2012; Streb and Zeeman, 2012; Pfister and Zeeman, 2016; Smith and Zeeman, 2020). Thus, it is believed that he has wealth of experience to achieve this feat and the outcome of the research will be credible. Also, the claim that the work was not peer viewed before it was presented for trial in Nigeria may be a little bit premature at the time due to the fact that the transgenic cassava had been tested in greenhouse for 3 years in ETHZ Biotechnology Lab in Zurich, Switzerland. As a result, it makes sense to test the transgenic cassava’s performance in a natural cassava growing environment, and the application was approved through the proper channels. Additionally, not all scientific discovery is peer-reviewed before patenting. The Nigerian government’s takeaway from this experience ought to be to fortify the nation’s scientific workforce or promote joint research endeavours between Nigerian scientists and their international counterparts in order to progressively employ plant biotechnology instruments to tackle issues concerning food security in the nation.

## Perspective and conclusion

In Nigeria, many people are involved in the direct production of various food crops. In fact, the country currently leads in the production of certain food crops globally, but this is usually due to increase in land conversion to crop production and not because of increase in yield/hectare. Production of other food crops is also suboptimal due to limiting factors like disease-infested planting material and susceptibility to biotic and abiotic stresses. While progress has been made using conventional breeding methods to develop crop varieties that are resistant, tolerant, or well adapted to both biotic and abiotic stress, this method is still limited in some ways. Techniques of plant biotechnology, such as genetic engineering and genome editing are increasingly becoming viable and sustainable options for improving our crops under certain conditions or circumstances. This review, therefore, provides general overview of the status of biotechnology in Africa with specific focus on Nigeria. Areas where biotechnological techniques will be most needed for crop improvements were identified and scientists can begin to take advantage of these novel techniques to provide lasting solutions to major problems, preventing Nigeria from reaching its full potential in crop production. Presently, the Nigerian government has enacted policies and regulations that favour responsible application and development of biotechnological products according to international best practice. Nigeria seems to be championing commercialization of biotech crops in Africa with recent approval of more genetically modified crops. Therefore, we recommend that governments in other African countries should take a cue from Nigeria by setting the stage for their countries to benefit from this technology towards ensuring food security and economic prosperity on the continent. Additionally, Nigerian government and start-up companies should also tap into immense potential of molecular farming for developing and production of subunit vaccines to bring about revenue and employment generation. The opposition from pressure groups against biotechnological application in Nigeria is largely due to the myth portraying the technology as foreign and not based on empirical evidences. For over two decades, several countries have benefited from sales and consumption of biotechnology products without any negative effects on the health of consumers.

## Figures and Tables

**Table:** 

**AAGEPF**	AUDA-NEPAD APET Genome Editing Policy Framework
**ACPOFS**	Africa Common Position On Food Systems Food Security
**APET**	African Panel on Emerging Technologies
**ARCN**	Agricultural Research Council of Nigeria
**AU**	African Union
**AUDA-NEPAD**	African Union Development Agency-NEPAD
**CASS**	Cassava Source-Sink
**CFT**	Confined field Trial
**CIAT**	International Center for Tropical Agriculture
**CRISPR/CaS**	Clustered Regularly Interspaced Short Palindromic Repeats/CRISPR-associated protein
**CGIAR**	Consortium Group for International Agricultural Research
**FAOSTAT**	Food and Agricultural Organisation Statistics
**GEd**	Genome/Gene editing
**GMO**	Genetically Modified Organism
**HDR**	Homology Directed Repair
**HE**	Homing Endonucleases
**MLN**	Maize Lethal Necrosis
**IITA**	International Institute of Tropical Agriculture
**OFSP**	Orange Fleshed Sweet Potatoes
**NABDA**	National Biotechnology Development Agency
**NACCIMA**	National Association of Chambers of Commerce, Industry, Mine and Agriculture
**NBMA**	National Biosafety Management Agency
**NBP**	National Biotechnology Policy
**NERICA**	New Rice for Africa
**NEWEST**	Nitrogen Efficient Water Efficient Salt Tolerant
**NHEJ**	Non-Homologous End Joining
**NRCRI**	National Root Crops Research Institute
**NVP**	National Vaccine Policy
**RNAi**	RNA interference
**SHESTCO**	Sheda Science and Technology Complex
**SSN**	Sequence Specific Nucleases
**TALEN**	Transcription activator-like Effector Nucleases
**VAD**	Vitamin A Deficiency
**WAAPP**	West Africa Agricultural Productivity Program
**WHO**	World Health Organisation
**ZFN**	Zinc Finger Nucleases
